# Rimming Plates

**Published:** 1869-05

**Authors:** J. P. H. Brown

**Affiliations:** Augusta, Ga.


### RIMMING PLATES.

BY J. P. H. BROWN, AUGUSTA, GA.

As there are many cases where a rim fitted nicely over the edges of the gums of single teeth will not only add to the beauty and finish of the work, but also to its strength, it is very necessary to know the best way of doing it. In block-work I regard such rim to be indispensable.

In giving my method of rimming plates I claim no originality, for others may use the same. It may not be the *quickest* way, but when all the manipulations are done with tact and skill, the results are every thing that can be desired. The quickest and easiest way is not always the best. The process, whether single or complex, which produces the best work should always be the one chosen. After grinding up the teeth so as to fit accurately the plate, and arranging them on wax just as you want them to set in the mouth, see that the tops of the gums are even and have sufficient bevel to be grasped by the rim. The tops of some may have to be ground off at an angle, say of forty-five degrees; others may not need any grinding. If there should be any little space at any point between the tops of the gums and plate, fill it up nicely and smoothly with wax. Now take some plaster and pour it on a piece of paper and spread to the depth of half an inch. Then take the plate and teeth off of the articulating model and imbed the teeth, cutting surfaces down into the plaster until it comes to within an eighth of an inch of the part of the gum to be covered by the rim. After the plaster has hardened it should be nicely trimmed around the gums, and a thin coat of shellac varnish applied. Place a rim of wax around on the inner side of the plate, and let it extend from a half inch to an inch above the edge of the plate. Fit a piece of thin tin plate to the plaster, gums and plate in front at the median line, so as to divide the mould for the plaster impression of the parts to be covered by the rim into two sections. Oil the plaster, gums, plate and wax, and then pour plaster on to one of the sections. All air bubbles must be expelled, for if any get into the face of the model it will be worthless. The plaster should be worked as thick as possible, and the outer edge of the model squared up with the spatula. Pour the other section in the same manner. After the plaster is hard remove carefully each section, and trim so that there will be no difficulty in removing from the sand. Make the dies of zinc and lead, and swage the rim of plate No. 28 or 29 of the American gauge, or of No. 4 or 5 of the English. Remove the plate from the wax by warming it, and let the teeth remain in the plaster. Trim some of the wax away so that the plate can be replaced to the teeth and removed without any difficulty. Put the plate to its place on the teeth. and adjust one of the halves of the swaged rim to the gums and plate. See that the rim fits the margin of the plate and covers the tips of the gums from a sixteenth to an eighth of an inch. Confine the rim to its position by wire clamps. Remove the plate to a piece of charcoal and solder. Treat the other half of rim the same way. Both sections of rim can be soldered on in a few minutes. If any little vacancies should be at any point between the rim and plate, fill with gold foil and then solder. Use no more solder than is necessary. No one but a bungling operator will run his solder into heaps nor all over his plate. After the teeth are soldered on, the rim can be burnished down so closely to the gums that it will be impossible to get the point of the smallest instrument between. If the plate is of twenty-one or twenty-two carats it will bear any number of heatings without springing a particle.

This way or that way of performing an operation does not matter so much, only so the results are of the *highest order*. Some of the most brilliant chemical experiments ever performed by Davy, Wollaston, or Faraday, were done by but little and simple apparatus. Where these careful men succeeded, thousands of others could not repeat the experiments with the same results with apparatus of the most approved and costly construction. You can teach a pupil the principles of drawing and painting and the laws of perspective; give him all the necessary materials, but unless he has the requisite brain development you will fail to make him an artist. A man may learn the principles of Dentistry as a science, but unless he has the mental organization necessary to enable him to apply "this science" to practice, all the private instruction, Dental lectures and itinerant practice can not make him a Dentist.

